# Responses of *Glossina fuscipes fuscipes* to visually attractive stationary devices baited with 4-methylguaiacol and certain repellent compounds in waterbuck odour

**DOI:** 10.1371/journal.pntd.0007510

**Published:** 2019-07-05

**Authors:** Njelembo J. Mbewe, Rajinder K. Saini, Janet Irungu, Abdullahi A. Yusuf, Christian W. W. Pirk, Baldwyn Torto

**Affiliations:** 1 International Centre of Insect Physiology and Ecology, Nairobi, Kenya; 2 Department of Zoology and Entomology, University of Pretoria, Hatfield, Pretoria, South Africa; 3 Pestinix, International Pest & Vector Control Specialists, Nairobi, Kenya; Yale School of Public Health, UNITED STATES

## Abstract

**Background:**

A blend of compounds (pentanoic acid, guaiacol, δ-octalactone and geranylacetone) identified in waterbuck (*Kobus defassa*) body odour referred to as waterbuck repellent compounds (WRC) and a synthetic repellent 4-methylguaiacol have previously been shown to repel tsetse flies from the morsitans group. However, these repellents have not been evaluated on palpalis group tsetse flies. In this study, we evaluated the effect of these repellents on catches of *Glossina fuscipes fuscipes* (major vector of human sleeping sickness) in biconical traps and on sticky small targets which are visually attractive to palpalis group flies. The attractive devices were baited with different doses and blends of the repellent compounds. We also assessed the effect of removal of individual constituents in the synthetic blend of WRC on catches of *G*. *f*. *fuscipes*.

**Methodology/Principal findings:**

The study was conducted in western Kenya on four islands of Lake Victoria namely Big Chamaunga, Small Chamaunga, Manga and Rusinga. The tsetse fly catches from the treatments were modeled using a negative binomial regression to determine their effect on catches. In the presence of WRC and 4-methylguaiacol (released at ≈2 mg/h and ≈1.4 mg/h respectively), catches of *G*. *f*. *fuscipes* were significantly reduced by 33% (*P*<0.001) and 22% (*P*<0.001) respectively in biconical traps relative to control. On sticky small targets the reduction in fly catches were approximately 30% (*P*<0.001) for both 4-methylguiacol and WRC. In subtractive assays, only removal of geranylacetone from WRC significantly increased catches (by 1.8 times; *P* <0.001) compared to the complete blend of WRC.

**Conclusions/Significance:**

We conclude that WRC and 4-methylguaiacol reduce catches of *G*. *f*. *fuscipes* at stationary visually attractive traps and suggest that they may serve as broad spectrum repellents for *Glossina* species. We recommend further studies to investigate the effects of these compounds on reduction of *G*. *f*. *fuscipes* attracted to human hosts as this may lead to development of new strategies of reducing the prevalence and incidence of sleeping sickness.

## Author summary

Tsetse flies are divided into three taxonomic groups: morsitans, palpalis and fusca. Flies from the morsitans and palpalis groups are the main vectors of trypanosoma parasites that cause human and animal African trypanosomiasis. The chemical 4-methylguaiacol and waterbuck (*Kobus defassa*), a known non-preferred host of tsetse, body odour blend (pentanoic acid, guaiacol, δ-octalactone and geranylacetone), have been shown to repel morsitans group species *G*. *pallidipes* and significantly reduce levels of animal African trypanosomiasis. However, these repellents have not been evaluated against other groups of tsetse, for example, those in the palpalis group. Here, we show that visually attractive stationary devices (biconical traps and sticky small targets) when baited with these repellent compounds and some of their blends significantly repel *G*. *f*. *fuscipes*, one of the important vectors of human sleeping sickness belonging to the palpalis group. The results provide the foundation for future studies of these compounds on their repellency of other *Glossina* species and their use in ‘push-pull’ strategies for manipulation of attraction to human and animal hosts and also in disease reduction strategies especially for riverine tsetse flies which are major vectors of parasites that cause sleeping sickness.

## Introduction

Tsetse flies (Diptera: Glossinidae) feed on blood and are biological vectors of African trypanosoma parasites that cause human and animal African trypanosomiasis [1]. They find their vertebrate hosts through olfactory and visual cues [2]. Beyond its visual range, the fly is activated by the odour from the host and orients upwind following the odour plume until it comes near the host where visual cues of colour, shape and size may elicit a landing response [2–5]. It is while taking a blood meal from a host that an infected tsetse fly transmits the parasites that cause African trypanosomiasis [6]. However, not all vertebrates found in tsetse fly habitats are fed on [7,8]. The differential preference of vertebrate hosts has been attributed to a combination of specific compounds found in the vertebrate’s body odour which could either attract or repel tsetse flies [2,4,8–10]. Consequently, research on identification of repellents to break the host-tsetse fly contact as a method of control against African trypanosomiasis (AT) has been ongoing since the 1970s [11]. Pioneer work on repellents showed that, humans are poorly attractive to *Glossina pallidipes* and *G*. *morsitans morsitans* which are tsetse species that belong to the morsitans group [2]. Variations in lactic acid concentrations in human odour were identified to be responsible for this repellency [12]. Since then, a number of synthetic and naturally occurring repellent compounds have been identified [9–11,13–15]. Among these are; acetophenone and 4-methylguaiacol which have been shown to reduce catches of *G*. *pallidipes* by 69% and 80% respectively [11,13], and δ-nonalactone which reduce *G*. *pallidipes* catches by 76% when used in attractant odour baited traps [15].

Furthermore some naturally occurring tsetse repellents found in the body odour of waterbuck (Bovidae: *Kobus defassa*), a non-preferred host were identified [7,8] including 15 compounds comprising of straight chain carboxylic acids (C_5_-C_10_), phenols (guaiacol and carvacrol), 2-alkanone homologues (C_8_-C_12_), geranylacetone and δ-octalactone [8,10]. A blend of all these compounds was found to significantly reduce catches of *G*. *pallidipes* in traps baited with odourant by 84% [10]. The blend of these compounds was reduced to a four component-blend [16] comprising pentanoic acid, guaiacol, δ-octalactone and geranylacetone referred to as waterbuck repellent compounds (WRC) which was found to reduce levels of animal African trypanosomiasis transmitted by *G*. *pallidipes* by 80% [16].

Most reports on tsetse fly repellents have been associated with important species belonging to the morsitans group. However, important tsetse fly species belonging to the palpalis group also responsible for human and animal African trypanosomiasis transmission in central and western Africa have received less attention. The important tsetse species belonging to the palpalis group include *G*. *fuscipes* subspecies, *G*. *palpalis* subspecies and *G*. *tachinoides*. Palpalis group tsetse species account for over 95% of transmissions of all human African trypanosomiasis (HAT) cases [17,18]. Among these, *G*. *fuscipes* subspecies with *G*. *f*. *fuscipes* having the widest distribution account for about 90% of transmissions in the HAT foci of central and west Africa [17–19]. Though they are opportunistic blood feeders, tsetse from the palpalis group have shown preferences to vertebrate hosts as observed from blood meal analysis [7,20]. For example, monitor lizards were consistently found to be the main hosts in Central African Republic, Kenya and Uganda accounting for about 40% of the blood meals [7]. Generally, tsetse flies from the palpalis group are reported to exhibit weak responses to host odours compared to flies from the morsitans group [5]. However, there is evidence from studies that have shown a general conservation of chemosensory gene families across five tsetse species which include *G*. *austeni*, *G*. *brevipalpis*, *G*. *pallidipes*, *G*. *m*. *morsitans* and *G*. *f*. *fuscipes* [21–24]. In this study, we hypothesised that repellents previously shown to be effective against morsitans group tsetse flies could also be repellent to palpalis group tsetse flies. Therefore, we evaluated the responses of *G*. *f*. *fuscipes* to visually attractive stationary traps baited with WRC and 4-methylguaiacol. We also assessed the effect of individual constituents of WRC on trap catches through subtractive assays.

## Materials and methods

### Study area

The experiments were carried out on four islands of Lake Victoria in western Kenya from April 2016 to December 2017. The Islands included: Small Chamaunga (latitude -0.431°, longitude 34.227°; surface area of about 0.2km^2^), Big Chamaunga (latitude -0.426°, longitude 34.227°; surface area of about 0.2km^2^), Manga (latitude -0.353°, longitude 34.253°; surface area of about 1km^2^) and Rusinga (latitude -0.358°, longitude 34.218°; surface area of about 43km^2^) [18,25,26]. Big and Small Chamaunga Islands are not inhabited by humans while Manga and Rusinga Islands are. The vegetation on the islands mainly consists of *Aeschynomene eraphyroxylon* (fresh water mangroves), *Lantana camara* (Tickberry) and *Dombeya* spp. (tropical hydrangea) [18]. These islands exclusively harbor *G*. *f*. *fuscipes* which mainly feeds on monitor lizards (*Varanus niloticus*) [18,26,27]. However, no case of sleeping sickness has been reported for the last 30 years in the study area [18].

### Capture devices and test compounds

Tsetse fly catches were made using biconical traps [28] and sticky small targets [29]. These were placed at sites that had either open or dense vegetation previously shown to have apparent fly densities of more than twenty flies per biconical trap per day [25]. The compounds: pentanoic acid, guaiacol, δ-octalactone and geranylacetone were blended in similar proportions (3:2:3:1) as found in waterbuck odour [8,10,16]. All individual compounds and blends of repellent compounds from waterbuck and 4-methylguaiacol were dispensed passively in natural environmental conditions from sealed polythene sachets (Audion Elektro, Derby, UK) with 0.125 mm thick walls, 50 mm × 75 mm in width and height placed next to the biconical trap and underneath the sticky small target [30]. All compounds evaluated were of 98–99% purity and sourced from ChemSamp Co, LLC, Trenton, USA.

### Observational study for release rates of test compounds

A series of subtractive assays to achieve blends without one constituent of WRC (Table 1) were prepared in sachets.

**Table 1:** Various treatments of subtractive assays of WRC and individual constituents.

Serial Number	Treatments
1	WRC
2	WRC without pentanoic acid
3	WRC without guaiacol
4	WRC without δ-octalactone
5	WRC without geranylacetone
6	No repellent
7	Pentanoic acid
8	Guaiacol
9	δ-octalactone
10	Geranylacetone

Three sachets, each containing WRC, blends that resulted from subtractive assays of WRC and individual constituents (Table 1) were subjected to field conditions. The weight of each dispenser was then taken every 24 hr for two days to come up with six replicates (S2 Table) of each treatment in order to determine the release rates. The average release rate of 4-methylguaiacol was obtained from nine sachets by measuring the difference in masses between freshly prepared sachets and their masses after 24 hr for three days (27 replicates in total; S2 Table) under field conditions.

In order to confirm the release of all compounds from the blends, a sachet containing either WRC or blends that resulted from subtractive assays were placed in a 700 ml glass bottle covered with aluminium foil tightly held to the bottle with two tight fitting rubber bands at room temperature. A pre-cleaned (through thermal desorption at 250°C for 30 min to remove any contaminants) 65 μm polydimethylsiloxane (PDMS) solid phase micro extraction (SPME) fibre (Supelco, Bellefonte, USA) was inserted through the aluminium foil into the bottle and the PDMS fibre exposed to the headspace for 5 min to adsorb the volatiles. Thereafter, the volatiles collected on the SPME fibre were subjected to GC/MS analysis. The fibre was manually inserted into the injection port of a 7890B Agilent gas chromatograph (Agilent Technologies, Wilmington, DE) coupled to an Agilent mass spectrometer (MSD 59977A, Agilent Technologies, Wilmington, DE) operated in split-less mode with the injector at 250°C to desorb the trapped volatiles for 2 min. The separation of compounds were done on an Agilent HP-5 MS capillary column (30 m × 0.25 mm id × 0.1 μm film thickness; Agilent Technologies, Santa Clara, US) using the following temperature programme: 35°C for 5 min, then raised at 10°C/min to a final temperature of 280°C and held for 10.5 min. Helium was used as the carrier gas at a constant flow rate of 1 ml/min. The compounds were detected using the electron ionisation mode (70eV; Ion source 230°C; quadrupole 150°C; mass scan range, 30–350 amu).

### Experimental design

Experiments where biconical traps were used ran from 8:00 to 18:00 hr [31] while those that used the sticky small target as the trapping device ran from 08:00 to 12:00 hr during the period when *G*. *f*. *fuscipes* is most active [27, 29]. The treatments were incorporated into a series of randomised block design experiments comprising groups of near or adjacent days at a site as different blocks [32]. Treatments were randomly allocated to days within these blocks. The number of blocks for each experiment which serve as replicates are shown in S1 Table. Tsetse fly catches were sexed and recorded for each treatment and experiment with freshly prepared repellents used for each experiment.

### Effective release rates of WRC and 4-methylguaiacol

Different release rates for the WRC and 4-methylguaiacol as treatments were achieved by varying the number of dispensers between one, two and four sachets per trap with the unbaited traps serving as controls. The number of sachets containing the compounds that effectively reduced both male and female catches of *G*. *f*. *fuscipes* in odourant baited biconical traps was used to bait sticky small targets to assess if the effect was similar to that observed in biconical traps.

### Effect of removal of individual constituents from WRC on catches of *G*. *f*. *fuscipes*

The blends that resulted from subtractive assays (two sachets), WRC (two sachets) and individual constituent (one sachet) of WRC served as treatments were compared to the control being a biconical trap alone without odour bait.

### Data analyses

All statistical tests were done with R version 3.2.5 [33]. Analysis of variance (ANOVA) and Student Newman Keuls (SNK) test were used for multiple comparisons of average release rates from single sachets of the individual constituents of WRC; resultant blends from subtractive assays and WRC. A negative binomial model was used to measure the effect of various treatments on the fly catch while taking into account the block and experimental day. Only the detransformed means (effects display) of treatments are reported and were obtained from the negative binomial regression using the “*effects*” package in R [34]. Statistical significance was considered at α less than 0.05.

### Ethics statement

Permission was given to undertake entomological gathering on Big Chamaunga and Small Chamaunga islands by the owners (International Centre of Insect Physiology and Ecology). Other entomological collection was done on public land. This study was conducted in conformity with the International Centre of Insect Physiology and Ecology ethical rules for animals.

## Results

### Release rates of test compounds

The average release rate from each polyethene sachet of WRC was 0.83 mg/h (95% CI: 0.65–1.01) while that of 4-methylguaiacol from each polythene sachet was about 1.4 mg/h (95%CI: 1.30–1.50). From single sachets of individual constituents, δ-octalactone had the lowest release rate (0.26 mg/h; 95% CI: 0.08–0.44), while pentanoic acid had the highest (3.83 mg/h; 95% CI: 2.29–4.01) (Table 2). For single sachets of the blends, WRC without pentanoic acid had the lowest release rate, whereas WRC without δ-octalactone had the highest release rate (Table 2). There were significant differences in the release rates from single sachets of the different blends and those of individual constituents of WRC (ANOVA, df_44_, F = 175.81, *P*<0.001). The overall release rate in mg/h of WRC without pentanoic acid or guaiacol was not significantly different from that of WRC (SNK: *P*>0.05; Table 2). However, when δ-octalactone or geranylacetone were removed from WRC, release rates differed significantly (SNK: *P*<0.05; Table 2).

**Table 2:** Average release rates of the repellent compounds from waterbuck dispensed from polyethene sachets.

Repellent compounds	Average release rates in mg/h (CI) n = 6
WRC	0.83 (0.65–1.01)^f^
WRC without pentanoic acid	0.80 (0.62–0.98)^f^
WRC without guaiacol	1.07 (0.89–1.24)^f^
WRC without δ-octalactone	3.08 (2.90–3.26)^b^
WRC without geranylacetone	1.48 (1.30–1.66)^e^
Pentanoic acid	3.83 (3.65–4.01)^a^
δ-octalactone	0.26 (0.08–0.44)^g^
Geranylacetone	1.75 (1.57–1.93)^d^
Guaiacol	2.47 (2.29–2.64)^c^

CI is 95% confidence interval. Average release rates with the same super script letter are not significantly different.

**Fig 1:**
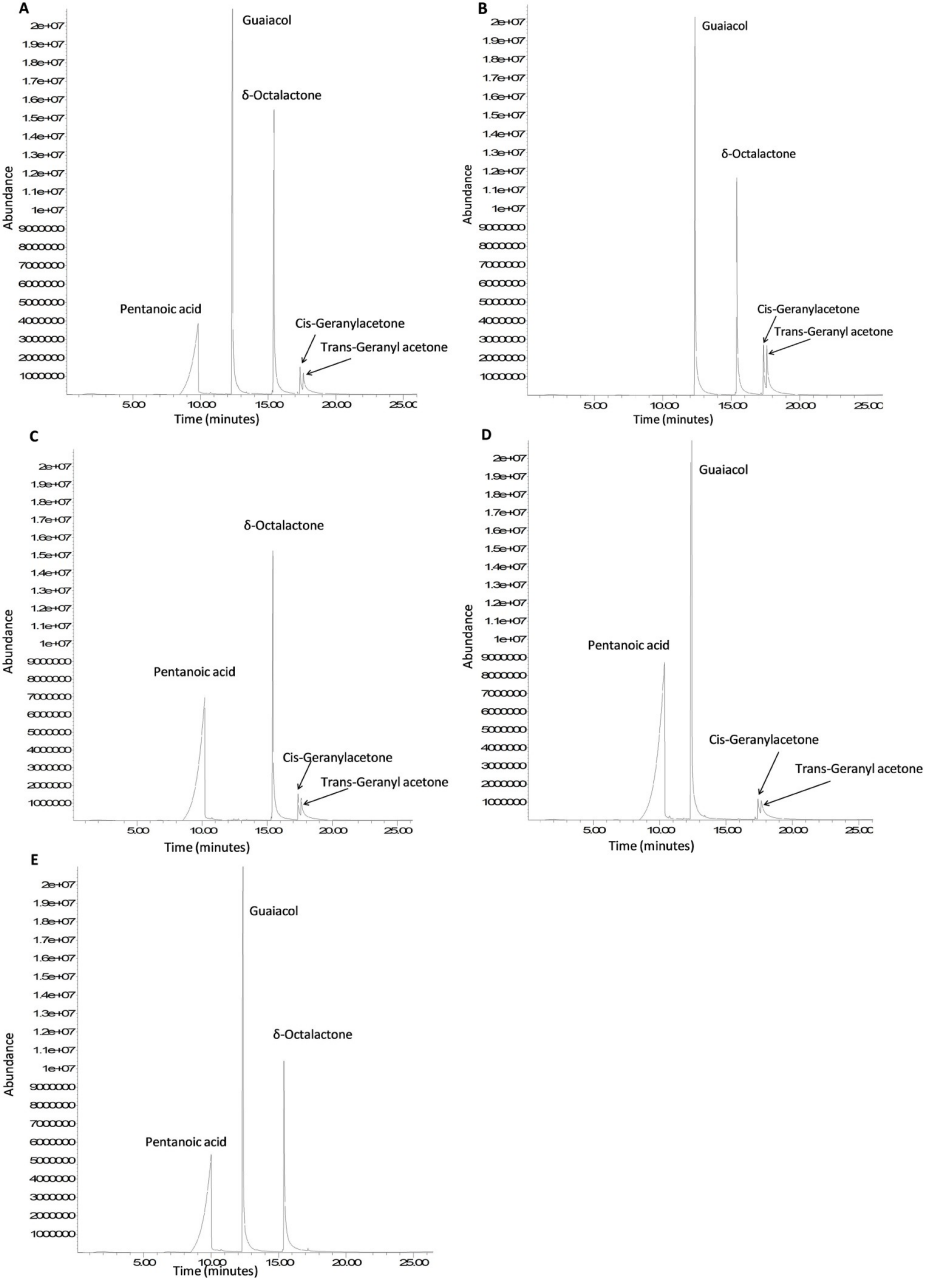
Total ion chromatograms. WRC (A), WRC without pentanoic acid (B), WRC without guaiacol (C), WRC without δ-octalactone (D) and WRC without geranylacetone (E).

Samples of sachets containing WRC and WRC without a specific constituent subjected to GC/MS confirmed that all the individual constituents were dispensed from the polyethene sachet dispensers as volatiles (Fig 1).

### Effective release rates of WRC and 4-methylguaiacol

**Fig 2:**
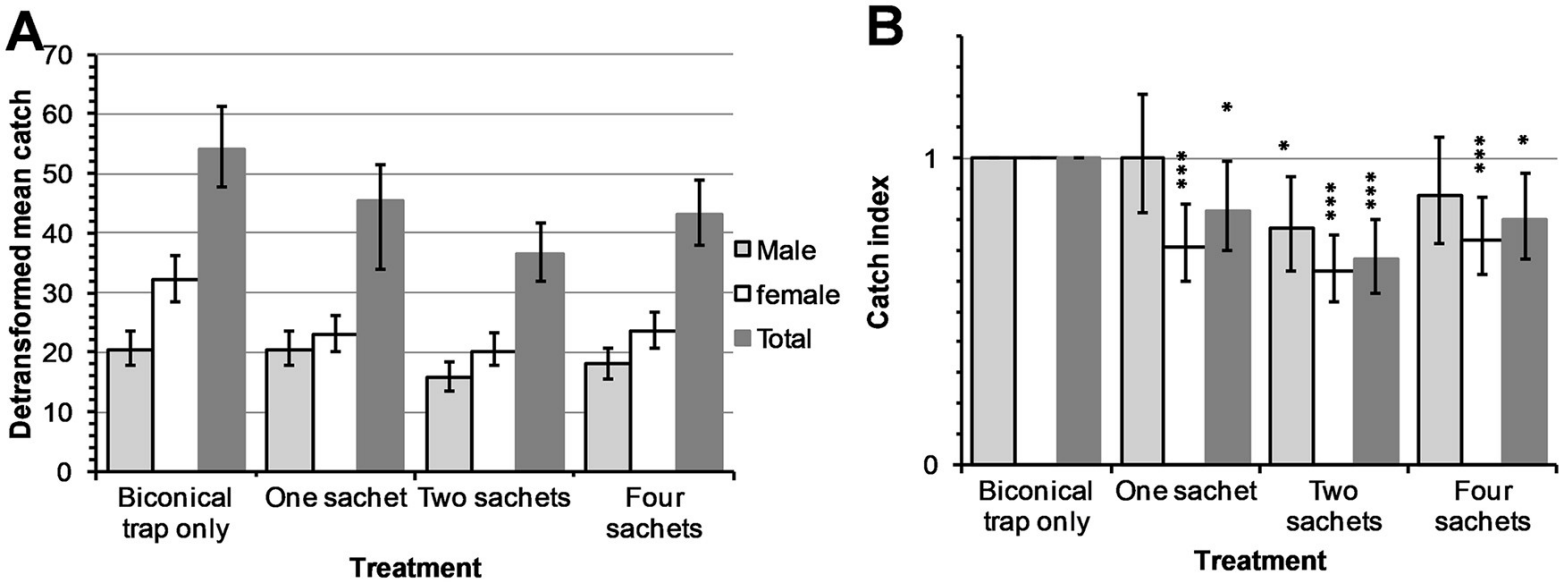
WRC dispensed from varying number of sachets at biconical traps. Detransformed means (A) and indices (B) of *G*. *f*. *fuscipes* catches Error bars indicate 95% CI of the detransformed mean (A) and catch index (B). *P<0.05 and ***P<0.001.

The total numbers of tsetse flies trapped in experiments with WRC were 3,983 of which 1,664 (41.8%) were males and 2,319 (58.2%) were females (S1 Table). The overall detransformed means of flies trapped in the control (biconical trap only) were higher than those collected from biconical traps with varying number of WRC dispensers as treatments (Fig 2A). The tsetse fly catches for both male and female *G*. *f*. *fuscipes* were significantly reduced when WRC was dispensed from two sachets at a biconical trap by 23% (95% CI: 6–37%; *P*<0.05) and 37% (95% CI: 25–47%; *P*<0.001) respectively and overall by 33% (95% CI: 20–44%; *P*<0.001). However, when WRC was dispensed from one and four sachets at biconical traps, only the female catches were significantly reduced (Fig 2B).

**Fig 3:**
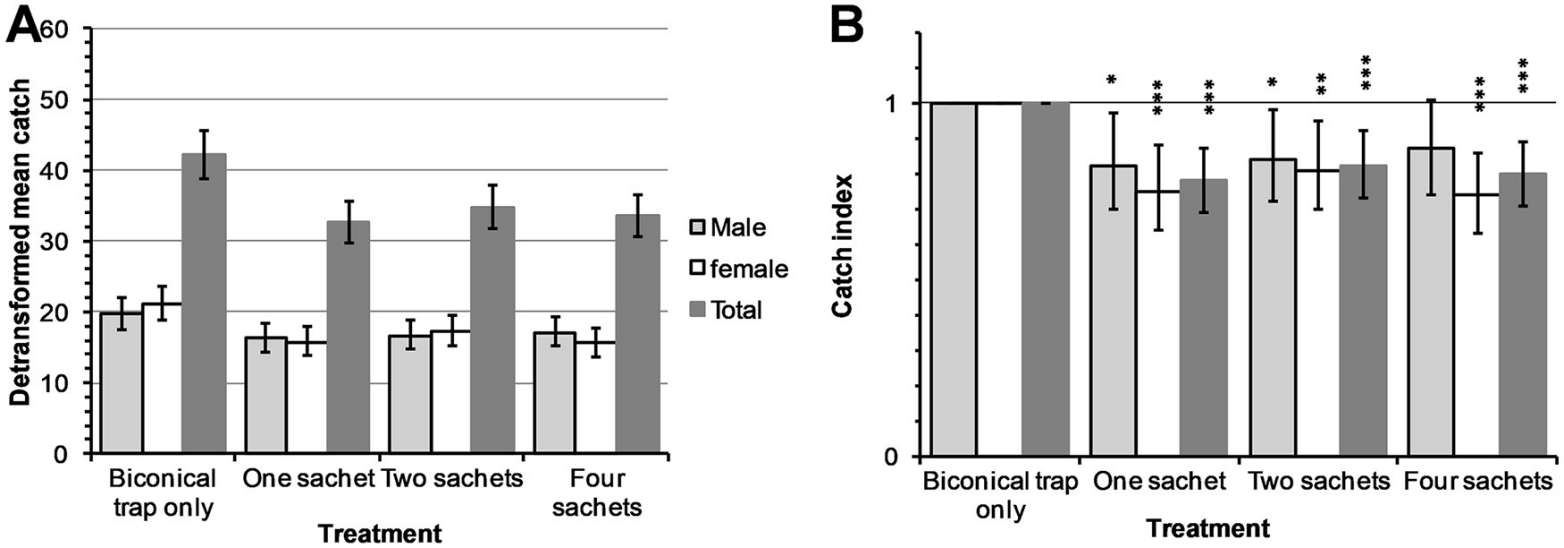
4-methylguiacol dispensed from varying number of sachets at biconical traps. Detransformed means (A) and indices (B) of *G*. *f*. *fuscipes* catches. Error bars indicate 95% CI of the detransformed mean (A) and indices (B) of tsetse fly catches. *P<0.05, **P<0.01 and ***P<0.001.

In experiments with 4-methylguaiacol a total of 2,589 tsetse flies were caught in traps comprising of 1,302 (50.3%) males and 1,287 (49.7%) females (S1 Table). The detransformed means of flies caught in the control (biconical trap only) were again higher than for traps with treatments (Fig 3A). Dispensing 4-methylguaiacol from one and two sachets significantly reduced catches of male *G*. *f*. *fuscipes* by 18% (95% CI: 3–30%; *P*<0.05) and 16% (95% CI: 2–28%; *P*<0.05) respectively while those of females were reduced by 25% (95% CI: 12–36%; *P*<0.001) and 19% (95% CI: 5–30%; *P*<0.01) respectively. Overall, when 4-methylguaiacol was dispensed from one and two sachets, the reduction in catches were 22% (95% CI: 13–31%; *P*<0.001) and 18% (95% CI: 8–26%; *P*<0.001) respectively. However, dispensing 4-methylguaiacol from four sachets only reduced female catches significantly (26%; 95%CI: 14–33%; *P*<0.001) (Fig 3B).

**Fig 4:**
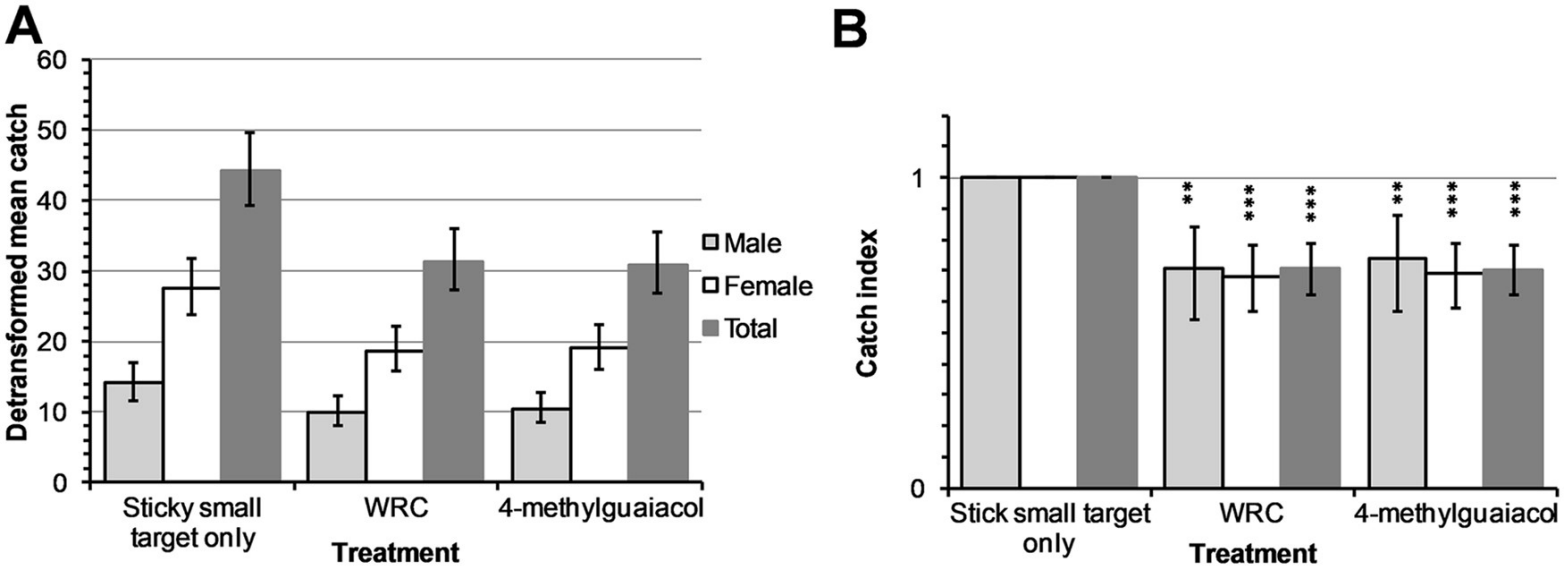
WRC and 4-methylguaiacol dispensed from two and one polyethene sachet respectively at sticky small targets. Detransformed means (A) and indices (B) of G. *f*. *fuscipes* catches. Error bars indicate 95% CI of the detransformed mean (A) and indices (B) of tsetse fly catches. **P<0.01 and ***P<0.001.

Two sachets of WRC and, a sachet of 4-methylguaiacol shown to be effective in reducing catches of both male and female *G*. *f*. *fuscipes* in previous experiments at biconical traps were dispensed from sticky small targets. This was done in order to test the effectiveness of the test compounds at different trapping device. The total number of tsetse flies caught in the experiment was 1,695 comprising of 610 (36.0%) males and 1,085 (64.0%) females (S1 Table). The results showed lower detransformed means (Fig 4A) and significant reductions in catch indices of both sexes of *G*. *f*. *fuscipes* compared to the control (Fig 4B).

### Effect of removal of individual constituents from WRC on tsetse fly catches

**Fig 5:**
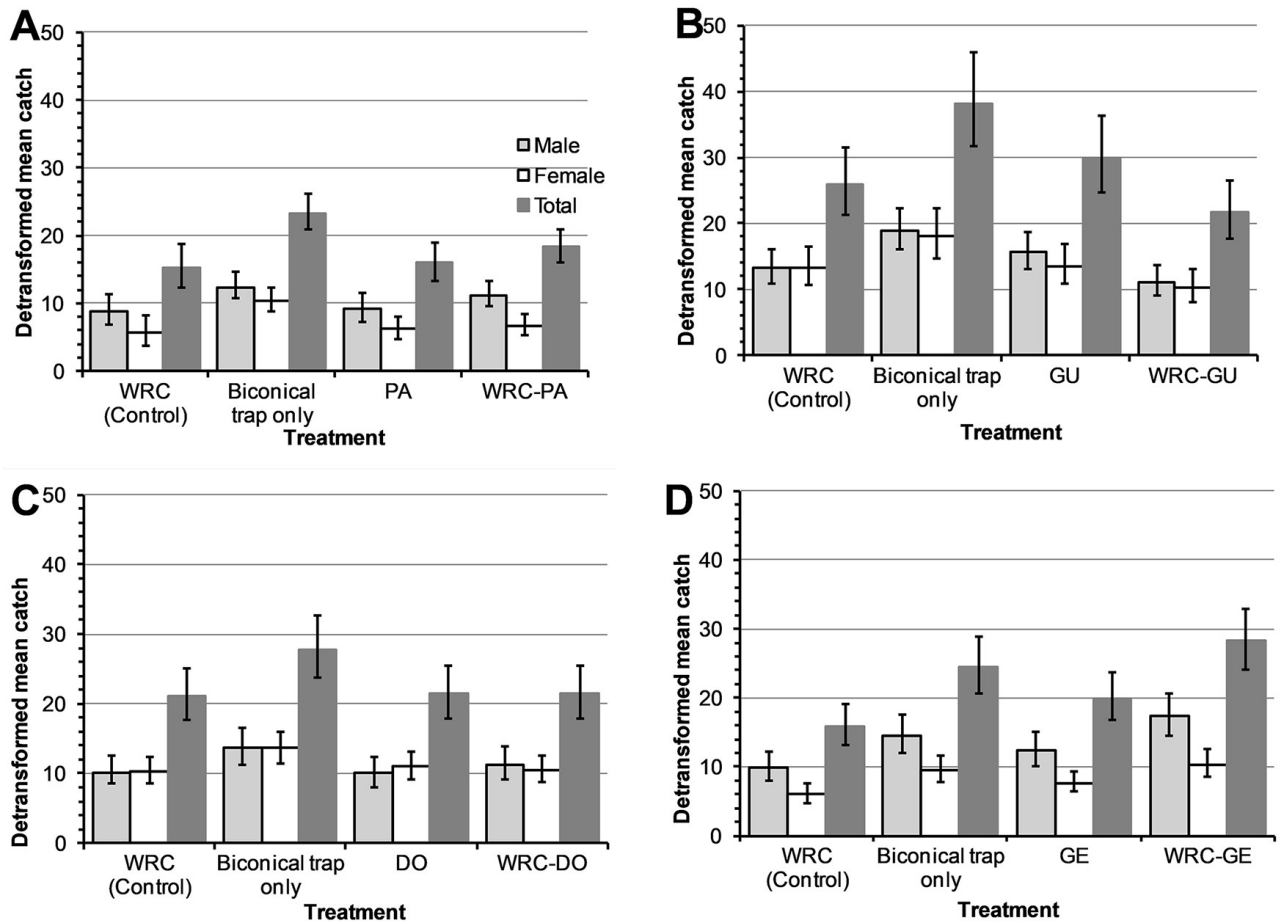
Mean catches of *G*. *f*. *fuscipes* in biconical traps from WRC subtractive assays. Traps baited with WRC without pentanoic acid (A), WRC without guaiacol (B), WRC without δ-octalactone (C) and WRC without geranylacetone (D). PA, GU, DO and GE represent pentanoic acid, guaiacol, δoctalactone and geranylacetone respectively. Error bars signify 95% confidence interval of the mean catch.

**Fig 6:**
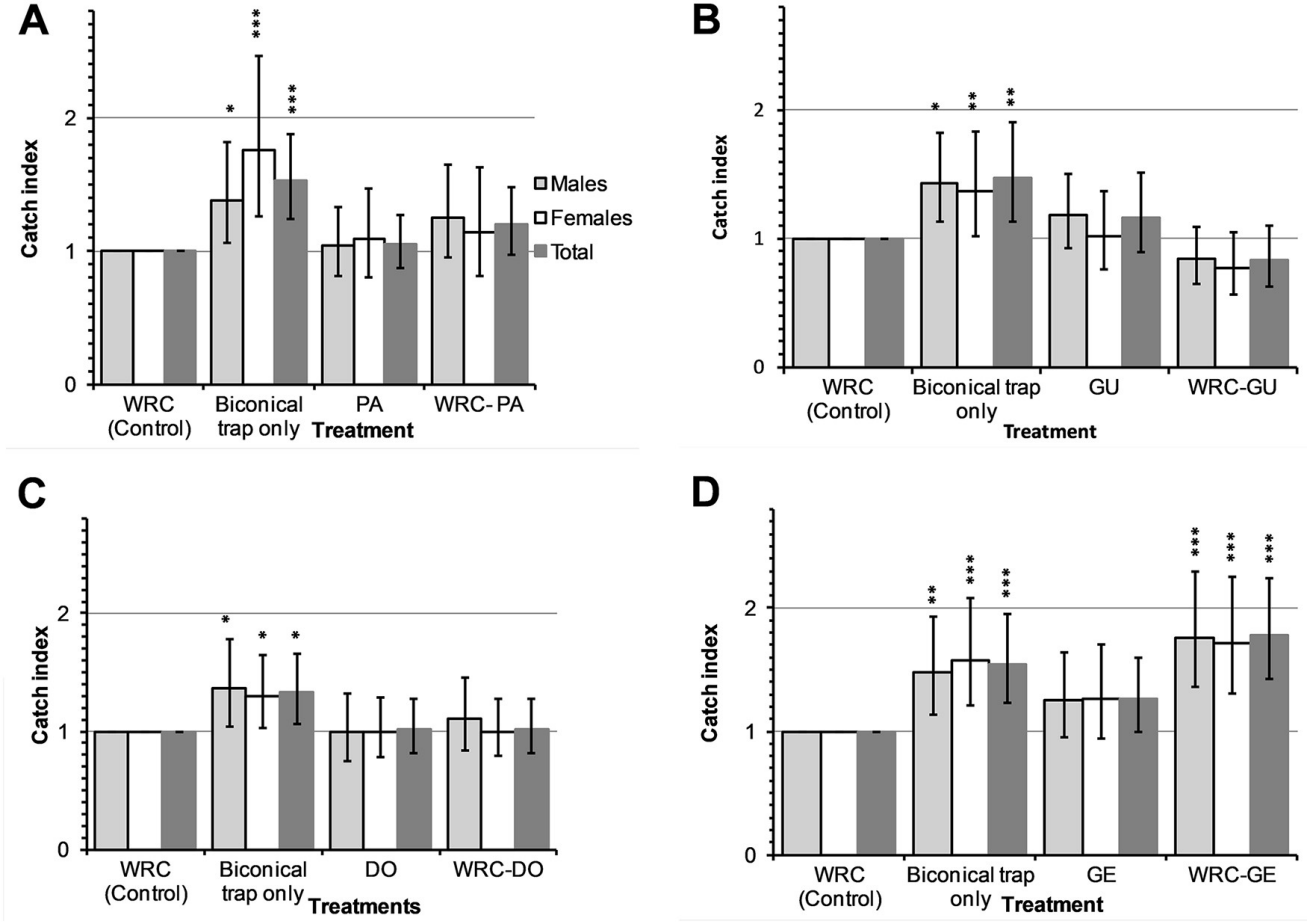
Percentage reduction in catches of *G*. *f*. *fuscipes* in biconical traps from WRC subtractive assays. Traps baited with WRC without pentanoic acid (A), WRC without guaiacol (B), WRC without δoctalactone (C) and WRC without geranylacetone (D). PA, GU, DO and GE represent pentanoic acid, guaiacol, δ-octalactone and geranylacetone respectively. Error bars signify 95% CI of the percentage reduction in catches. *P<0.05, **P<0.01 and ***P<0.001.

During these experiments, a total of 5,489 *G*. *f*. *fuscipes* were caught comprising of 2,923 (53.3%) males and 2,566 (46.7%) females (S1 Table). The removal of pentanoic, guaiacol or δ-octalactone from WRC neither lowered the overall detransformed mean nor reduced the catches of *G*. *f*. *fuscipes* compared to biconical traps baited with WRC (P>0.05; Figs 5A–5D and 6A–6D). Dispensing WRC without geranylacetone from two sachets significantly increased the catch of male and female *G*. *f*. *fuscipes* by 1.76 times (95%CI: 1.36–2.29 times; *P*<0.001) and 1.71 times (95% CI: 1.31–2.25 times; *P*<0.001) respectively compared to WRC.

## Discussion

Assessing the responses of tsetse flies from the palpalis group to synthetic compounds and natural odours that repel flies from the morsitans group is important as it may lead to development of novel African trypanosomiasis control methods. Such control methods could be effective by reducing host-vector contact particularly in areas with low density and infection rates in tsetse populations, such as those of the HAT foci in central and west Africa [5]. In this study, we report responses of *G*. *f*. *fuscipes*, a tsetse species from the palpalis group, to visually attractive stationary traps baited with WRC and 4-methylguaiacol.

We observed that both male and female catches of *G*. *f*. *fuscipes* in biconical traps were reduced at specific dispensing rates of WRC (2 dispensers approximately 2.0 mg/h) and 4-methylguaiacol (1 or 2 dispensers approximately 1.4 and 2.8 mg/h respectively), indicating that they are true repellents as defined by Dethier et al. [35]. The repellency of WRC and 4-methylguaiacol to *G*. *f*. *fuscipes* was confirmed by the reduction of catches on sticky small targets baited with these compounds. These results seem to indicate that the release rates of odorants influence the responses of *G*. *f*. *fuscipes*. This conforms to findings from other studies which showed that it was only at release rates of 100 mg/h of lactic acid that *G*. *pallidipes* were repelled compared to a release rate of 10 mg/h [13]. In addition, other blood feeding Diptera such as mosquitoes, also demonstrate responses that are dependent on odorant concentrations [36].

We also observed a differential sex response when WRC was dispensed from a single or four sachets (approximately 1.0 or 4.0 mg/h respectively) with only female catches being reduced. A similar observation with 4-methylguaiacol was made with only female catches reducing when it was dispensed from four sachets (approximately 5.6 mg/h). Related differential sex responses have been reported with *G*. *pallidipes*, a tsetse fly from the morsitans group, to constituents of human odour where the repellent effect was greater for females than males [2]. This could be an indication that female *G*. *f*. *fuscipes* are more sensitive to repellent odours than males. Consistent to the observed differential sex response to odorants in our study, is the finding by Otter et. al. [37] where female *G*. *f*. *fuscipes* had higher electroantennogram responses than males. Nevertheless, there is need to investigate further the role of this differential sex response in the biology of the fly as it could be exploited for development of control methods that target female *G*. *f*. *fuscipes*.

Furthermore, the observed repellency of *G*. *f*. *fuscipes* to WRC and 4-methylguaiacol could be exploited for “push-pull” disease control strategies where the repellents could be used to “push” the flies towards stationary visual attractive (“pull”) devices that eventually kill the flies. The consistent response to repellent odours in *Glossina* species observed in this and other studies provides support to a study that showed that there is a general conservation of chemosensory gene families across five tsetse species that includes *G*. *f*. *fuscipes* and *G*. *pallidipes* [10, 11, 21].

The reduction in *G*. *f*. *fuscipes* catches of ~33% by WRC we observed is less than ~84% reported for *G*. *pallidipes* [10]. This variation could be due to the differences in formulation of WRC, where in our case, hexanoic acid was not added as a constituent. However, the repellency of hexanoic acid was reported not to be significantly different from that of pentanoic acid for *G*. *pallidipes* [10]. Additionally, the previous study used traps baited with odour attractants as the controls (reference) [10]. In this study, the use of traps without odour attractants as controls could also explain the differences in reduction in fly catches. However, the reduction in *G*. *f*. *fuscipes* catches of ~22% by 4-methylguaiacol is also less than ~70% reported for *G*. *pallidipes* at traps without odour attractants [11]. This could be an indication that *G*. *f*. *fuscipes* is less sensitive to 4-methylguaiacol than *G*. *pallidipes*. Even though this is consistent with other reported observations that tsetse flies in the palpalis group show, markedly weaker responses to host odours compared to those from the morsitans group [5], further studies to ascertain why this is the case are needed.

Despite being dispensed from sachets of relatively consistent measurements, WRC without δ-octalactone or geranylacetone had significantly higher release rates compared to WRC. Clearly, indicating that in blends, the relative diffusion of different constituents across the walls of the polyethene sachet dispensers and subsequent evaporation from the surface is not only influenced by their respective vapour pressures, but also by the presence of other components in the blend [10]. This is further supported by the observed significant variation in release rates of the individual constituents of WRC.

Our results also indicate that when geranylacetone is removed from WRC; the catch of the resultant blend (pentanoic acid, guaiacol and δ-octalactone) increased by 1.8-fold, showing less potency in repellency. This suggests that geranylacetone may be playing an important role to the overall repellent effect of WRC to *G*. *f*. *fuscipes*. In other dipteran vectors, repellence by geranylacetone has been implicated in the differential attraction of humans to various species of mosquitoes and *Culicoides* midges [38, 39]. Geranylacetone has also been reported to enhance the repellency of a mixture of ammonia and lactic acid against mosquitoes [38]. Conversely, pentanoic acid enhances attraction at flow rates of 100ml/min of a mixture of ammonia and lactic acid [40].

Interestingly, fly catches in traps baited with the blends that result after removal of pentanoic acid (geranylacetone, guaiacol and δ-octalactone), guaiacol (geranylacetone, pentanoic acid and δ-octalactone) or δ-octalactone (geranylacetone, guaiacol and pentanoic acid) from WRC (pentanoic acid, guaiacol, δ-octalactone and geranylacetone) did not significantly differ with those from traps with WRC. Additionally, the catches at traps with the individual constituents did not also significantly differ from those of WRC. These results suggest that the individual constituents could substitute WRC as repellents at biconical traps. Pentanoic acid and guaiacol have been shown to reduce tsetse fly catches of *G*. *pallidipes* at Epsilon traps but not significant effect on the feeding efficiency of the fly [13]. This indicated that what works at traps may not work to protect hosts against tsetse fly bites. With *G*. *fuscipes* being a major vector of HAT [18] and evidence that it can be repelled from traps baited with synthetic and allomonal compounds from waterbuck odour, we recommend further studies that will evaluate these compounds to protect human hosts and dwellings.

Most studies that compare trap catches of tsetse flies exposed to several treatments of odorants use Latin square design experiments [10, 11, 13, 15]. This is because it can control for variation of fly catches due to the trap site and day at study design stage [41]. One of the important assumptions of the Latin square design is that all trapping sites should have the same sort of vegetation [41]. However, in this study trapping sites were in both open and dense vegetation. Therefore, we used a randomised block design where the site was blocked and treatments were randomly assigned to days as experimental units in each block. This way the randomisation controlled for unknown confounds while known confounds were addressed during statistical analysis by accounting for the block and day of the experiment. Additionally, our study did not consider the effect of age of the polythene sachet dispensers as it could affect the release rates of the repellents [42]. However, for each experiment, freshly prepared repellents dispensers where used. This could have minimised the effect of age of the sachets on the release rates of repellents. Studies with other Dipteran vectors, such as mosquitoes have used fabric-based dispensers for odorants [43]. Further studies should explore how these dispensers could also perform for tsetse flies. Furthermore, on account that the release rates reported were not concurrently obtained from experiments that compared catches of tsetse flies from traps baited with odorants, it is possible that these could be biased. However, the setting and odorant dispensers used to obtain the release rates were similar to those used in the field experiments of comparing the effect of odorants on trap catches of tsetse flies. Thus, we are confident that these could reflect the actual field release rates of the odorants.

In conclusion, the present study has shown that WRC and 4-methylguaiacol released at specific rates reduced catches of both sexes of *G*. *f*. *fuscipes* in unbaited traps, an indication that they are true repellents. It also showed that sex of *G*. *f*. *fuscipes* could play a role in its responses to repellents. Additionally, geranylacetone seems to play an important role in the overall repellency of WRC. Furthermore, individual WRC constituents: pentanoic acid, guaiacol, geranylacetone and δ-octalactone repel *G*. *f*. *fuscipes* just as well as WRC at biconical traps. Therefore, we recommend further studies to evaluate the repellency of 4-methylguaiacol, WRC and its individual constituents against *G*. *f*. *fuscipes* in the presence of hosts as it may lead to the development of novel control methods especially in HAT foci.

## Supporting information

S1 Table Fly catches for each experiment. (XLSX)

S2 Table Collected data that was analysed. (XLSX)
